# Coronary Artery Disease and Its Risk Factors Status in Iran: A Review

**DOI:** 10.5812/kowsar.20741804.2286

**Published:** 2011-09-15

**Authors:** M Ebrahimi, S M R Kazemi-Bajestani, M Ghayour-Mobarhan, G A A Ferns

**Affiliations:** 1 Cardiovascular Research Center, Avicenna (Bu-Ali) Research Institute; 2 Department of Cardiology, Mashhad University of Medical Sciences, Mashhad, Iran; 3 Radboud University Nijmegen Medical Center, Nijmegen, The Netherlands; 4 Department of Nutrition and Biochemistry, Mashhad University of Medical Sciences, Mashhad, Iran; 5 Unite´ de Recherche Ge´ne´tique Cardiovasculaire, Nancy-Universite´, Faculte´ de Pharmacie, France; 6 Institute for Science and Technology in Medicine, University of Keele, Staffordshire, UK

**Keywords:** Coronary artery disease, Prevalence, Risk factors, Metabolic syndrome, Iran

## Abstract

**Background:**

Coronary artery disease (CAD) is a leading cause of mortality, morbidity, and disability in the world. The high prevalence and morbidity associated with CAD in Iran is one of the most pressing health problems.

**Methods:**

We have reviewed the status of CAD and prevalence of its traditional and novel risk factors based on the published papers in recent years that may have an impact on the rate of CAD in Iran. Evaluation of current impact of metabolic syndrome in Iran was the other aim of this review, as it contributes to pathogenesis of coronary artery disease. We reviewed all PubMed indexed related studies. Some evidences from national articles which were published by the Ministry Of Health and Medical Education Research Council Certified Medical Journals of Islamic Republic of Iran were also included.

**Results:**

The prevalence of CAD, coronary risk factors and metabolic syndrome in Iran is higher than Western countries and similar to some Middle East countries. There are limited data with regard to novel coronary risk factors in Iran.

**Conclusion:**

Primary and secondary prevention of CAD including life style modifications and dietary interventions strongly recommended in Iranian population.

## Introduction

Coronary artery disease (CAD) is a condition in which atherosclerotic plaque builds up within the wall of the coronary arteries leading to narrowing and the clinical manifestations of acute coronary syndrome including angina and myocardial infarction. It is a leading cause of mortality, morbidity, and disability in the world.^1^ Recent data indicate that the Iranian adult population has a high prevalence of CAD risk factors.^2^ The high prevalence and morbidity associated with CAD in Iran is one of the most pressing health problems.^3^ In this article, we have reviewed the current status of CAD prevalence and its risk factors based on the published papers in recent years which may impact on this problem in Iran.

## Materials and Methods

### Study Selection

Med-Line (1970-2010) were searched using the subsequent keywords: CAD, risk factors, prevalence, incidence, hypertension, smoking, dyslipidemia, hyperlipidemia, diabetes mellitus, obesity, metabolic syndrome, addiction, putative coronary risk factors, prooxidant-antioxidant balance, C-reactive protein, heat shock protein, trace elements, homocysteine, lipoprotein and Iran. Searches were not restricted by language or study format.

We selected all studies that reported the prevalence of CAD and/or risk factors. All different types of description of CAD in the investigations were considered (e.g. based on electrocardiogram, angiography). Having reviewed full text of references, studies which estimated the prevalence of CAD in a nonrandom sample or in a small sample size less than 100 were excluded. Subsequently, the quality of studies was evaluated according to items related to their objectives, population or sample characteristics, inclusion/exclusion criteria, usage of the same mode of data collection for all subjects and its validity, clearly described findings, interval estimations, and appropriate data analysis methods. Furthermore, duplicated citations and those studies that assessed the prevalence in children and infants were excluded. For more clarification, we also evaluated some few articles which were published by Ministry of Health and Medical Education Research Council Certified Medical Journals of the Islamic Republic of Iran by using Iran medex website. The main focus of this review was CAD and its risk factor status in Iranian population.

### Prevalence of CAD and Atherosclerosis

Sarraf-Zadegan (1999) *et al*. reported among the target sample of 6,470 men and women aged 35-79 years who were randomly selected from 80 random clusters in Isfahan that the overall prevalence of CAD based on the Rose Q and/or ECG was 19.4% and was significantly higher among women 21.9% than men 16.0%;^4^ this differs from other global populations.

Sadeghi *et al*. (2006) reported the prevalence of CAD, in 6498 people aged above 35 years, based on the Rose questionnaire and Minnesota coding to be 37.5% in women and 22.2% in men;^5^ this is clearly a very high prevalence if true for the whole Iranian population. Fakhrzadeh *et al*. (2008) in a population-based study from Qazvin (Central Iran) found that the age-adjusted prevalence of possible myocardial infarction, ischaemic ECG changes and angina pectoris were 4.2%, 36.8% and 2.2% respectively.^6^ Hadaegh *et al*. (2009) reported on a sample population of 5984 men and women aged > or = 30 years and coded by Minnesota criteria that the aged-adjusted prevalence of CAD was 21.8% (22.3% in women and 18.8% in men).^7^

We have also reported different findings from Iran that indicated the high rate of atherosclerosis and atherosclerotic related diseases and CAD in Iran.^8-16^ These Iranian articles also proved high levels of all traditional risk factors among Iranian with manifestation of atherosclerotic diseases.^8-16^ A summary of the reported CAD prevalence in Iranian population based on different studies is shown in Table 1.

**Table 1:** Prevalence of CAD in several Iranian investigations.

Authors	Years of investigation	Selected population	Age of selected population	Method of evaluation	Prevalence
Sarraf-Zadegan (1999) *et al*., ^4^	1999-2002	6,470	35-79	Rose Q and/or ECG	19.4%
Sadeghi *et al*., (2006) ^5^	2000-2001	6498	>35	Minnesota coding	30.2%
Fakhrzadeh *et al*., (2008)^6^	2007	846	≥25	Recorded prevalence of possible myocardial infarction, ischemic ECG changes, angina pectoris	4.2% 36.8% 2.2%
Hadaegh *et al*., (2009)^7^	2006-2008	5984	≥30	Minnesota coding	21.8%
Vahdat *et al*., (2007)^93^	2003-2004	1,754	25-66	Minnesota coding	12.6%
Rezaei Ghaleh *et al*., ^94^	2003-2004	5539	>30	According to ECG findings, the age-adjusted prevalence of probable and possible CAD	1.1% 9.3%

CAD, coronary artery disease

## Traditional Coronary Risk Factors

A study published in 2004 reported the prevalence and distribution of hypertension, cigarette smoking, dyslipoproteinemia, diabetes mellitus, and obesity in 15005 subjects, aged three years and over, selected by cluster random sampling in the Tehran urban district13 between February 1999 and August 2001. In adults (>20 years old), 78% of men and 80% of women had at least one traditional CVD risk factor. The percentage of adult women with two or more risk factors was significantly greater than for men. The prevalence of diabetes mellitus (DM), hypertension, obesity, high total cholesterol (TC), low high density lipoprotein cholesterol (HDL-C), high trigycerides (TGs), and smoking was 9.8, 20.4, 14.4, 19.3, 32, 5.3, and 22.3%, respectively and finally they concluded that the prevalence of cardiovascular risk factors among population in Tehran was high; particularly the presence of high total cholesterol, low HDL cholesterol levels, and high waist to hip ratio.^17^ Kelishadi *et al*. (2005) designed a project to evaluate the cumulative prevalence of atherosclerotic cardiovascular disease risk factors in a representative sample of Iranian adolescents (1,000 girls and 1,000 boys, ages 11-18 years) selected by multi stage-random cluster sampling from urban and rural areas of three cities in Iran and demonstrated the prevalence of physical inactivity, dyslipidemia, smoking, high blood pressure and obesity (body mass index > 95th percentile) were 66.6, 23.7, 8.7, 5.7 and 2.2%, respectively. Among subjects studied, 79.1% had at least one and 24.6% had two cardiovascular disease risk factors.^18^ A summary of traditional coronary risk factors status of Iranian population is shown in Table 2.

**Table 2:** Prevalence of traditional coronary risk factors in Iranian population.

Authors	Risk factor	Number of Selected population	Age of selected population	Applied description	Prevalence
Azizi *et al*., (2003)^24^	Dyslipidemia	6246	6246	Total cholesterol values between 200-239 mg/dL Total cholesterol values ≥ 240 mg/dL	31% 24%
Sharifi *et al*., (2008)^25^		2941	>20	Low HDL-C Hypertriglyceridemia (>150 mg/dL) Increased total cholesterol (>200 mg/dL) The combination of hypertriglyceridemia and low HDL-C	73% (M: 63%; F: 93.3%) 40.6% 35.4% 9.9% 3.6% 5.5%
Azizi *et al*.,^26^ Azimi-Nezhad *et* *al*., (2009)^28^ Sadeghi *et al*., (2007)^30^	Diabetes mellitus	595 717 3,778 12,514	>30 between 15 and 64 >19	Fasting bloodGlucose≥126mg/dL Fasting blood Glucose≥126mg/dL Fasting blood Glucose≥126mg/dL	3.6% 5.5% 6.7% (Urban area) and 5.3% (Rural areas)
Karimi *et al*., (2009)^33^ Esteghamati *et* *al.*, (2006)^31^		10,622 * 514**	Mean :58.75 ± 9.72 years -	Fasting blood Glucose≥126mg/dL Fasting blood Glucose≥126mg/dL	35% 30%
Haghdoost *et al*., (2008)^36^	Hypertension	93,661	30–55 >55	Review article: SBP>140mmHg, DBP>90mmHg	23% 50%
Esteghamati *et* *al*., (2008)^37^		***6.6 million Iranians	25-64	SBP≥140mmHg, DBP≥90mmHg	25%
Sadeghi *et al*., (2004)^38^ Kelishadi *et* *al*.,(2006)^39^		12494 21,111	>19 6-18	SBP≥140mmHg, DBP≥90mmHg Systolic, diastolic as well as systolic or diastolic hypertension according to the Second Task Force study 95th percentile cut-off points	Men (15.6%) and Women (18.8%) systolic, diastolic as well as systolic or diastolic: 4.2, 5.4 and 7.7%, respectively
Emami *et al*., (2003)^44^	Smoking (cigarette)	11,801	>=15	Daily smokers	10.6% (22% were males and 2.1% females)
Fotouhi *et al*., (2009)^45^		4565	>=15	Daily smokers	11.9% [20.6% of the male and 2.9% of the female participants (P<0.001].
Sarrafzadegan *et al*., (1999)^46^		2,200	19-70	Daily smokers	11% [26.0% of the male and 1.4% of the female participants (P<0.001].
Sarrafzadegan (2004)^47^		2626	>19	Self-reported smoking: Serum nicotine level	M:18.7% F:1.3% M:21.2% F:6.7%
Kelishadi *et al*., (2006)^50^		11,966	11-18	Self-reported cigarette smoking	14.3% [18.5% of the male and 10.1% of the female participants (P<0.001].

*This study was performed on consecutive patients undergoing elective coronary artery bypass graft; ** This study included patients with unstable angina or myocardial infarction; ***Estimation for these numbers of Iranians; SBP, systolic blood pressure; DBP, diastolic blood pressure;M, male; F, female

### Dyslipidaemia

Dyslipidemia is present at a high frequency in Iran.^19-24^ The study by Sharifi *et al*. (2008) is an investigation of 2941 individuals in Iran, and reported that the most prevalent abnormality was a low serum HDL-C (< 50 mg/dL in females < 40 mg/dL in males; 73% including 63% for men and 93.3% for women); hypertriglyceridemia (>150 mg/dL) was the second most prevalent abnormality (40.6%); increased total cholesterol (>200 mg/dL) was observed in 35.4% of the subjects and the combination of hypertriglyceridemia and low HDL-C was observed in 9.9% of the population.^25^ Ebrahimi *et al*. (2009) also showed that low serum HDL-C concentration was one of the strongest factors that independently associated with CAD in Iranian population.^10^ It has been reported in several investigations that dyslipidema was significantly more common in Iranian patients.^10,11,14-16^

### Diabetes Mellitus

The prevalence of type 2 DM ranges from 1.2% to 14.6% in Asia, 4.6% to 40% in the Middle East, and 1.3% to 14.5% in Iran.^26-28^ The age-standardized death rate related to cardiovascular diseases and diabetes is estimated to be higher than 400 per 100,000 in Iran.^29^ However, Sadeghi (2007) *et al*. reported the total prevalence of DM to be 6.7% and 5.3% in urban and rural areas and 5.4% and 7.1% in males and females, respectively in Iran, while study was performed on 12,514 subjects from 3 cities in the central part of Iran on participants over the age of 19 years.^30^ Diabetes mellitus is highly prevalent in the Iranian population with established CAD.^10,14-16,31^ Jang-horbani *et al*. (2006) indicated that there is a high prevalence of CAD among the Iranian type 2 diabetic patients.^32^ In a study of 10,622 consecutive patients undergoing elective coronary artery bypass grafts (CABG) from 2001 to 2005 revealed that over one third of these patients had DM.^33^ The patients with left main coronary artery disease were more likely to be male, older, and have diabetes mellitus or dyslipidemia in Persian population.^34^ Esteghamati *et al*. (2006) proved that 30% of the patients with acute coronary syndrome had DM in Iran.^31^ A study with a sample population of more than 10,000 persons clarified that the prevalence of dyslipidaemia in diabetics was 88.9% in Iran.^35^

### Hypertension (HTN)

There is strong proof that in developing countries such as Iran, the rapid increase in the prevalence of HTN has started more recently. A systemic review by Haghdoost *et al*. (2008), who worked on 29 eligible previous reports with a total sample size of 93,661 subjects and also access to the results of a very large national survey, which reported the prevalence of HTN in 27 provinces of Iran estimated the overall prevalence of hypertension in 30-55 and > 55-yearold population was approximately 23% and 50%, respectively.^36^ Esteghamati *et al*. (2008) reported that the prevalence of HTN and pre-HTN was high in Iran. They also reported that approximately 25% or 6.6 million Iranians aged 25-64 years had hypertension; additionally 46% or 12 million Iranians aged 25-64 years had prehypertension.^37^ The prevalence of high blood pressure in men and women was 15.6% and 18.8% respectively in Iran according to a cross sectional investigation of was performed in three cities of Iran on participants over 19 years at 2002 for more than 12500 sample population.^38^ The prevalence of elevated blood pressure was 0.8% in the study of 6038 (3280 boys 2758 girls) apparently healthy students in Iran in 2007.^39^ It was shown that HTN was significantly higher in Iranian patients with confirmed CAD compared to normal population.^10,11,14-16^ Findings of a 12 years study from Iran clarified that the age-adjusted incidence rate of hypertension was 22% lower among insulin-treated than non-insulin-treated type 2 diabetes mellitus clinic attainders and it was greater with older age.^40^ Ghanbarian *et al*. from the Tehran Lipid and Glucose study (comprising 2479 men and 3060 women aged > or =30) reported that prevalence of ECG-defined MI in hypertensive persons was two-fold higher than in normotensives.^41^

### Smoking

During the last several years in Iran, widespread studies have been conducted on smoking, of which National Health and Disease Survey, as the most extensive one, reported a decrease in smoking from 14.6% in 1991 to 11.7% in 1999.^42,43^ Large-scale national studies evaluated 11,801 cases in Iran, the "Tehran Lipid and Glucose Study", showed the prevalence of daily cigarette smoking to be 10.6%.^44^ The results of two other Iranian studies also indicated that the prevalence of smoking in Tehran was about 11%.^45,46^

Sarrafzadegan *et al. *(2004) showed other work which represented the prevalence of self-reported smoking among Iranian men and women aged 19 years and above which was 18.7% and 1.3%, respectively, compared to 21.2% and 6.7% based on serum nicotine level. Nearly 10.6% and 14.6% of claimed non-smoker girls and boys were classified as current smokers by serum nicotine level.^47^ Gharipour *et al*. (2008) in an Iranian study comprising 1,625 smokers and 3,948 non-smokers, with a mean age of 38.07±14.85 years, reported that serum LDL-C and triglycerides were higher in smokers than in non-smokers.^48^

Kelishadi *et al*. (2004) performed a cross-sectional study among 1950 students, aged ll-18, selected by multi-stage random sampling from three cities in Iran and finally revealed these findings: The mean values of total-and LDL-C were higher in smokers and their HDL-C was lower than non-smokers. The mean systolic and diastolic blood pressures were higher in smokers than non-smokers. The smokers had higher BMI than non-smokers. The mean food consumption frequency was lower for fruits and vegetables and higher for fat/salty snacks and fast foods in smokers than non-smokers.^49^ This altered nutritional status might be due to lower educational levels of smokers. However, the exact reason is not clear. Kelishadi *et al*. (2006) conducted other research which was performed among 11,966 school students, ages 11-18 years, selected by multi-stage random cluster sampling from 20 provinces in Iran. The prevalence of self-reported cigarette smoking was 14.3%, with a higher prevalence in boys than in girls and in high school than in middle school students. Overall, 42.2% of smokers were daily smoker, and the rest were occasional smoker. The mean age of the first attempt to smoke was 13.2±2.5 years with no significant gender difference but was significantly lower in daily than in occasional smokers. The place of the first attempt to smoke was mostly in friend parties (41.6%) and traditional teahouses (23.2%) for boys and the family parties (37.1%) and the traditional teahouses (27.4%) for girls. The higher school level and having a tobacco user at home significantly increased the likelihood of smoking in adolescents, with the highest odds ratio found for having a smoker sister in girls, and having a smoker brother in boys.^50^

## Metabolic Syndrome (MetS)

MetS is characterized by a clustering of clinical and metabolic features that include high triglycerides, low HDL-C, high blood pressure, impaired glucose tolerance, visceral adiposity, and insulin resistance.^51^ Aziminezhad *et al*. reported that the age-adjusted prevalence of MetS in Khorasan Province of Iran according to the three definitions was 39.9% [Adult Treatment Panel III (ATP III); male: 29.1% and female: 50.4%], 40.5% [International Diabetic Federation (IDF); male: 26% and female: 54.5%], and 40.5% [American Heart Association (AHA); male: 30% and female: 50.1%], respectively. Among all participants, 38.9% (male: 22.7% and female: 52.9%) had MetS by all three definitions.^12^ They also reported that the highest prevalence of MetS was found within the sixth decade of life (ATP III: 58.4%, IDF: 57.5%, AHA: 59.7%; and 43.8% by all three definitions).^12^

According to the first phase of Isfahan Healthy Heart Program (IHHP), the prevalence of the MetS in non-pregnant women by ATP III criteria was approximately 21.9% in adults in central Iran.^52^ Kelishadi *et al*. (2008) carried out a cross-sectional study on 3694 subjects with age ≥ 19 years in Isfahan and proved that prevalence of MetS was 19.8% in females with normal BMI, 48.1% in overweight females and 63.2% in obese females. In males, corresponding values were 3.7%, 18.0% and 40.1%.^53^ Hadaegh *et al*. (2007) [(cross-sectional study, the study population consisted of a representative sample of 1737 males and 1707 females aged ≥ 20 years with normal body mass index (BMI) (18.5-24.9 kg/m2 for both genders); according to the ATP III guidelines] showed that the overall prevalence of the MetS in normal-weight men and women was 9.9% and 11.0% respectively.^54^ In a study of 2309 adults demonstrated that prevalence of MetS gradually rose with increasing grades of obesity, from 31.9% in the non-obese to 69.0% in the morbidly obese according to the IDF criteria and from 31.2% to 62.1% according to the ATPIII criteria.^55^ A total of 622 high school adolescents participated in a cross-sectional study in Mashhad and the findings were approximately 6.5% of all and 45% of obese subjects met the criteria for the metabolic syndrome.^56^

Much recent argument about the MetS had properly raised questions about its definition, its clinical impact, and even querying its existence as a clinical entity. Ebrahimi *et al. *(2009) mentioned that it appears that within an Iranian population, the presence of MetS defined by either the ATP III or IDF criteria failed to identify individuals with established, angiographically defined CAD.^10^ Sharifi *et al*. (2009) in an investigation from urban population of the west of Iran reported that metabolic syndrome was present in 23.7% of subjects (based on ATP III description), the prevalence was 23.1% in men and 24.4% in women. The prevalence increased from 7.5% in the population younger than 30 y to 45.6% in ages more than 50 years. Most of those with metabolic syndrome had three components of the syndrome (75.6%), 170 subjects (24.4%) had four and none had five components simultaneously.^57^ A summary of metabolic syndrome prevalence in the Iranian population is shown in Table 3.

**Table 3:** Prevalence of metabolic syndrome in Iranian population.

Authors	Number of Selected population	Age of selected population	Prevalence
Azimi-Nezhad *et al*., (2007)^12^	2353	15–65	ATPIII: 39.9% (male: 29.1% and female: 50.4%) IDF: 40.5% (male: 26% and female: 54.5%) AHA: 40.5% (male: 30% and female: 50.1%)
Mousavi *et al*., (2009)^52^	6331 non- pregnant women	>20	ATP III: 34.2%
Kelishadi *et al*., (2008)^53^	3694	≥19	ATP III : 19.8% in females with normal BMI, 48.1% in overweight females and 63.2% in obese females. In males, corresponding values were 3.7%, 18.0% and 40.1%.
Hadaegh *et al*., (2007)^54^	3444	≥20	ATP III: in normal-weight men and women : 9.9% and 11.0% respectively
Mirhosseini *et al*., (2009)^56^	622	15-17	ATP III:6.5%
Ebrahimi *et al*., (2009)^10^	1707 (431: candidate of angiography and 1276 subjects were drawn from the same local population but were not suspected of having CAD)	33-80	ATP III: 45.5% IDF: 48.4%
Sharifi *et al*., (2009)^57^	2941	>20	ATP-III: 23.7%

ATP III, Adult Treatment Panel-III; IDF, International Diabetic Federation; AHA, American Heart Association

## Obesity

Siavash *et al*. (2008) explored the association between obesity and coronary angiography findings of 591 patients in Isfahan and showed that the prevalence of CAD was significantly higher in abdominal obese patients than in patients without abdominal obesity.^58^ Nematy *et al*. (2009)^59^ in a study of about 2000 free living elder Iranian population (≥ 60 years) of Khorasan-Razavi Province proved that 28.9, and 11.7% of the total population were overweight and obese, respectively. They also indicated that the prevalence of central obesity was higher among Iranian elder women than men (63.1 vs. 18.6%, respectively).^59^ Hajian-Tilaki *et al*. (2007) conducted a population-based cross-sectional study with a sample of 1800 women and 1800 men with respective mean ages of 37.5±13.0 and 38.5±14.2 years of urban population aged 20-70 years living in the north of Iran and reported that the overall prevalence rates of obesity and overweight were 18.8% and 34.8% respectively.^21^ The overall prevalence rate of central obesity was 28.3% in the study of Hajian-Tilaki *et al*.^21^ The rate of obesity in women was significantly higher than men in northern Iran.^21^ Sajjadi *et al*. (2008) also evaluated a general population of 3940 Iranian subjects and indicated higher prevalence of obesity in females compared with males.^60^

The increased prevalence of obesity in Iranian women with cardiovascular disease has been shown in previous studies.^61-64^ Maddah *et al*. (2007) showed that obesity and central obesity in Iranian women with CAD (43.5% and 88.5%) were more prevalent than Iranian men with CAD.^62^ Chinikar *et al*. (2006) showed that advancing age and diabetes were independent predictors for development of CAD in Iranian overweight and obese women with documented CAD.^63^

Recently concerns arise about the increased prevalence of obesity in Iranian adolescents. Ghergerehchi *et al*. (2009) showed a 69.58% prevalence of dyslipidemia in overweight and obese children and adolescents of Tabriz city aged 4 to 18 years.^65^ Moadab *et al*. (2010) reported that among 7554 students from Isfahan (48.7% boys and 51.3% girls), 9.34% were overweight and 5.3% were obese.^66^ Maddah *et al*. (2010) carried out a study among randomly-selected 2,577 urban school girls aged 12-17 years from north of Iran and proved that the overall prevalence of overweight and obesity in this population was 18.6% and 5.9% respectively.^67^

## Addiction

Drug addiction is a social problem in Iran. The association between opium and opium derived drugs and cardiovascular diseases have been shown in some Iranian studies. Masoomi *et al*. (2010)^68^ have reported that drug abuse is an independent predictive risk factor for development of deep vein thrombosis in Kerman, Iran. Iranmanesh (2008) from relatively the same region (Rafsanjan, a city from Kerman Province) reported 45.7% opium addiction in the patients with ischemic stroke.^69^

Unfortunately, there is an incorrect general consideration between some Iranian cardiovascular patients that opium has a protective effect against cardiovascular diseases. This is one of the reasons that we may find higher frequency of addiction among Iranian cardiovascular patients.

Shirani *et al*. (2010) in an investigation of a total of 1,339 Iranian patients who were candidates of angiography proved that opium was not cardioprotective and also did not protect carotid arteries against atherosclerosis progression.^70^

Sadeghian *et al*. (2007)^71^ performed an investigation among the 2405 Iranian admitted patients in angiography ward with mean age of 57.5±10.3 years and established the 13.4% prevalence of opium consumption in all patients and 19.7% in men.^71^ Sadeghian *et al*.^71^ findings indicated a direct relation between opium consumption and angiography-defined CAD. Strong association between opium use and severity of CAD was also proved in Iranian cardiac patients.^71^

Sadeghian *et al*. (2010) carried out an study among 940 younger patients (men < 45 y/o and women <55 y/o) who underwent coronary angiography and concluded a significant association between angiography-defined CAD presence and opium consumption in men.^72^

Asgary *et al*. (2008) showed the adverse effect of opium addiction on atherogenic risk factors in Persian patients.^73^ According to Iranian studies, it is important to note that an association between opium addiction and CAD presence did not prove a direct relationship unless confounding factors were taken into account (e.g. education, socioeconomic status etc); It may be that people who tale drugs were poorer and that they were also at risk of CAD. Further studies which look at all confounding factors are required to define the exact role of opium addiction in CAD development.

Karam *et al*. (2004) also revealed the exacerbating role of opium addiction in increasing blood glucose in the Iranian patients with non-insulin-dependent diabetes mellitus.^74^ Azod *et al*. (2008) also proved no profit of opium consumption on improving the blood sugar status of diabetic patients.^75^ However the results of Kouros *et al*. (2010)^76^ investigations were not in accordance with previous Iranian studies.^76^ Kouros *et al*. (2010) showed the lower fasting blood sugar of the addicted persons who had consumed heroin or opium for more than two years compared with the control group.^76^ This is also likely to be confounded by nutritional status. It is well known that opium will have an effect on appetite and therefore weight. These confounding factors should be considered for future Iranian studies.

Sadeghian *et al*. (2009) retrospectively analyzed a total of 4,398 Iranian patients who had undergone isolated CABG and finally demonstrated opium dependency of 15.6% among patients.^77^ Sadeghian *et al*. (2009) proved the adverse effects of opium dependency on CABG outcomes.^77^ Najafi *et al*. (2009) showed the failed influence of opium dependency on improvement of quality of life in Iranian patients with the history of CABG.^78^

## Putative Coronary risk factors

Several investigations have been conducted in Iranian population on several putative coronary risk factors.

### Prooxidant-Antioxidant Balance (PAB)

There is a report from an Iranian sample population that determined the in patients with angiographically defined CAD and showed that the PAB value may be a cardiovascular risk factor.^26^ It may also indicated that a heightened state of oxidative stress following acute coronary syndrome and suggested that the PAB value might be considered as a cardiovascular risk predictor to estimate the extent of oxidative stress in Persian ethnicity.^14^

### C-Reactive Protein (CRP)

CRP is an acute phase reactant. The relationship between serum CRP concentrations and cardiovascular events provides one form of evidence linking the inflammatory response and risk of vascular disease.^79^ Kazemi-Bajestani *et al*. (2007)^13^ reported that serum high sensitive (hs)-CRP is an independent predictor of angiographically defined CAD in an Iranian population. Measurement of the serum hs-CRP level may improve risk stratification among patients suspected of having CAD. The strong correlations between serum hs-CRP with LDL-C and smoking may be due to the putative pro-inflammatory effects of these two parameters. The association with serum triglycerides may be indirect and related to insulin resistance and adiposity.^13^ However Nabipour *et al*. (2008) have reported that elevated CRP is significantly correlated with diabetes in general population of the northern Persian Gulf.^80^ Ghayour-Mobarhan *et al*. (2007) confirmed that serum CRP concentrations increased in patients with classical coronary risk factors in Iranian population, and that they might be modulated by dietary cholesterol.^81^

### Heat Shock Protein (Hsp)

Cells respond to a variety of environmental stresses by rapidly expressing a family of proteins called the Hsp. Ghayour-Mobarhan *et al*. (2009) highlighted the role of HSP-65 and 70 and smaller HSPs, such as HSP-27, in atherogenesis.^82^ Jafarzadeh A *et al*. (2008) reported that patients with ischemic heart disease had higher Chlamydial Hsp-60 in comparison with normal individuals.^83^

Ghayour-Mobarhan *et al*. (2008) reported that in Iranian population serum antibody titers to Hsp-27 raised and fell rapidly after the onset of acute coronary syndrome, and may be an early marker of myocardial ischemia as patients with myocardial infarction or unstable angina both had high titres.^84^

### Trace Elements Status 

Trace element status has been investigated as a possible contributory factor to atherosclerosis.

Ghayour-Mobarhan *et al*. (2009) conducted a study of a group of 2233 individuals, 15-65 years of age were recruited from residents of the Great Khorasan Province in northeast of Iran and reported a significant associations between serum trace element concentrations and several coronary risk factors, including calculated 10 years' coronary risk scores.^23^ Parizadeh *et al*. (2009) reported that mean serum selenium concentrations were not significantly different between patients with and without CAD and the control group.^15^

### Homocysteine

Hyperhomocysteinaemia is a novel CAD risk factor. Some of the Iranian population based studies also confirmed the association between serum homocysteine levels and presence of CAD.^85-87^ Kazemi *et al*. (2006) proved a linear correlation between serum homocysteine levels and the numbers of coronary artery involvement in Iranian individuals without any traditional risk factors.^87^

However, other Iranian reports have failed to demonstrate the relation between higher incidences of cardiovascular disease among patients with higher serum homocysteine levels.^88,89^ Nabipour *et al*. (2007) performed a cohort study of random sample of 1699 men and women aged ≥ 25 years (48.9% males, 51.1% females) and found hyperhomocysteinaemia (>14 micromol/l) 50.8% of the Iranian subjects.^89^ Nabipour *et al*. did not show an association between hyperhomocysteinaemia and CAD after adjusting of sex and age.^89^

Sadeghian *et al. *(2006) showed that hyperhomocysteinemia is an independent risk factor for angiography-defined CAD in young male patients (below 45 years old).^86^ Sadeghian *et al*. (2006) reported that hyperhomocysteinemia was not a risk factor of CAD development in Iranian females.^86^

### Lipoprotein a [Lp (a)]

A large body of evidence showed the association between increased serum levels of Lp (a) and development of CAD. Iranian investigators also confirmed the important role of serum Lp (a) in development and severity of CAD in several Iranian population-based studies.^90,91^ Rasouli *et al*. (2008) following a multivariate analysis , after adjusting for major risk factors, showed a significant and independent association of Lp (a) with the prevalence of CAD in Iranian subjects.^90^ Boroumand *et al*. (2008) conducted a cross-sectional study on 826 patients who underwent angiography and concluded that Lp (a) serum concentration is an independent risk factor for CAD development in the Iranian population particularly at the ages below 55.^91^ Boroumand *et al*. also demonstrated a direct relationship between severity of coronary atherosclerosis and serum Lp (a).^91^

This review indicates that the Iranian population has a higher prevalence of CAD in comparison to other regions of the world. Rapid changes in life style in Iran may have resulted in a decrease in levels of physical activity, and alteration in pattern of healthy nutrition. Genetically susceptibility and increase in the rate of all traditional cardiac risk factors might be the essential cause of high prevalence of CAD in Iran. Other prospective investigations are definitely required to show a useful pattern of morbidities and mortalities of CAD in Iran. The comprehensive statistical survey about incidence, mortality, out-of-hospital cardiac arrest, hospital discharges and ambulatory care visits may provide a more comprehensive picture of the current status of CAD in Iran. CAD is the single largest killer of American males and females. A significant difference between the prevalence of CAD between Iran and USA may exist; therefore intensive emergent preventive programs are required to reduce the prevalence of CAD in Iran.

The findings of this review based on several published articles revealed that different types of dyslipidemia are common among Iranian adults, in particular a high prevalence of low HDL-C in Iranian population that may be associated with obesity. Analytical methodologies for the measurement of HDL-C used in Iran need to be fully standardized, and would require appraisal by Iranian supervisors of laboratory measurements. Genetic and environmental factors may also help to explain the high prevalence of lower HDL-C in Iranian population. According to report of Ebrahimi *et al. *(2009)^10^ that low HDL-C is an independent predictive factor of CAD in Iranian population this point should be considered.

The prevalence of other features of dyslipidemia in Iran (e.g. hypertryglyceridemia) was also higher than the report of AHA.^92^ Controlling the major health disorder of dyslipidemia in Iran should be taken into detailed consideration for primary, secondary and tertiary prevention of CAD.

The prevalence of DM in Iranian population is almost the same as reported in Western societies.^92^ However developing countries, particularly in south Asia, are witnessing a rapid increase in DM. All reports from Iranian population confirmed at least 10% smoking in Iranian adults that seem high. However the interpretation of Kelishadi *et al*. report^50^ that 14.3 of the subject between 11-18 year old smoke cigarettes is not easy for us. This rate seems very high that is even higher that the prevalence in adults. High prevalence of smoking in very young age Iranian population is a very important warning point that sufficient attentions should be paid in this regard. These data should be attributed to a number of factors, including differences in population demographics, differing levels of tobacco control programs and policies, and variations in tobacco industry marketing and promotion.

The prevalence of MetS in Iranian population is high based on different articles. This high prevalence of MetS shows a high clustering of cardiovascular risk factors in Iranian population.

In several investigations, the prevalence about 40% has been reported. Ebrahimi *et al*. (2009)^10^ reported that the proportion of patients with MetS, defined by either the IDF or NCEP-ATP III, was not significantly different between three groups: those with angiographically defined CAD (case group) and those with a normal angiogram (control group) and reference group (subjects with no previous history of any major disease). Regarding the increased prevalence of obesity in Iranian women with cardiovascular diseases,^61-64^ special public health interventions are highly recommended. According to Persian studies it seems that obesity in females also increased in female adolescents. Therefore, further approaches should focus on obesity management in all age groups of Iranian females. Specially, Iranian elderly women in menopause ages due to the lack of hormonal protection are at high risk of obesity-related cardiovascular morbidities.^61-64^ Our review of Iranian investigations demonstrated a considerably high prevalence of opium consumption between Iranian cardiovascular patients.^71,78^ Some of Iranian studies did not prove any improving effects for opium use^70,75^ and most of them showed the influence of opium consumption on presence and exacerbation of cardiovascular diseases and their risk factors.^68,69,71,77^ According to high prevalence of opium consumption among cardiovascular patients and lack of knowledge of these patients about the adverse effects of opium and other opium-derivates, intensive programs should be performed by physicians for management of opium-dependency in these patients in Iran. However, if the patients are already dependent to opium, due to the immediate adverse effects of opium withdrawal, precise collaborations between cardiologists, psychiatrists and family physicians should be considered for tapering the opium consumption with comprehensive methodology.

Recent attentions of Iranian investigators for analyzing the novel coronary risk factors are very interesting. However, prospective studies demonstrating the clinical utility of these approaches are limited. These novel coronary risk factors have recently come to the fore as potential solutions to the challenge of detecting high-risk individuals for primary prevention. Although studies of serum markers of inflammation provide substantial insight into the pathophysiology of atherothrombosis in Iran, the clinical utility of measuring these markers remains uncertain in Iranian population.

There are two limitations that need to be acknowledged and addressed regarding the present study. The first limitation concerns not investigating all national Iranian articles. We only used little evidence of national published articles by using Iranmedex website.

Other main limitations of our study were that the most of the major investigations regarding CAD and its risk factors in Iran published from three area of Iran: Isfahan, Tehran and Mashhad. Therefore, it might not reflect the current status of Iranian CAD from other parts. Further national prospective studies may be helpful for more clarification. However, we started a cohort of cardiovascular risk factors in Mashhad area, which is a 15 years prospective study and hopefully the early results will publish very soon.

## Conclusion

The prevalence of CAD and coronary risk factors in Iran is higher than Western countries and similar to some Middle East countries. There are limited data in regard to novel coronary risk factors in Iran. The definition of some coronary risk factors such as MetS should be modified in Iranian population and some biochemical markers such as measuring HDL-C should be reevaluate in Iran. Primary and secondary prevention of CAD including life style modifications and dietary interventions strongly recommended in Iranian population for preventing of increasing and decreasing CAD in Iran.
